# Neurovascular coupling in patients with type 2 diabetes mellitus

**DOI:** 10.3389/fnagi.2022.976340

**Published:** 2022-09-01

**Authors:** Antonietta Canna, Fabrizio Esposito, Gioacchino Tedeschi, Francesca Trojsi, Carla Passaniti, Irene di Meo, Rita Polito, Maria Ida Maiorino, Giuseppe Paolisso, Mario Cirillo, Maria Rosaria Rizzo

**Affiliations:** Department of Advanced Medical and Surgical Sciences, University of Campania “Luigi Vanvitelli”, Naples, Italy

**Keywords:** type 2 diabetes mellitus, functional MRI, neurovascular coupling, cognitive impairment, cerebral blood blow, brain network

## Abstract

Functional and metabolic neural changes in Type 2 diabetes mellitus (T2DM) can be associated with poor cognitive performances. Here we analyzed the functional-metabolic neurovascular coupling (NVC) in the brain of T2DM patients. Thirty-three patients (70 ± 6 years, 15 males) with recent T2DM diagnosis and 18 healthy control (HC) subjects (65 ± 9 years, 9 males) were enrolled in a brain MRI study to identify the potential effects of T2DM on NVC. T2DM patients were either drug-naive (*n* = 19) or under treatment with metformin (*n* = 14) since less than 6 months. Arterial spin labeling and blood oxygen level dependent resting-state functional MRI (RS-fMRI) images were combined to derive NVC measures in brain regions and large-scale networks in a standard brain parcelation. Altered NVC values in T2DM patients were correlated with cognitive performances spanning several neurological domains using Spearman correlation coefficients. Compared to HC, T2DM patients had reduced NVC in the default mode network (DMN) and increased NVC in three regions of the dorsal (DAN) and salience-ventral (SVAN) attention networks. NVC abnormalities in DAN and SVAN were associated with reduced visuo-spatial cognitive performances. A spatial pattern of NVC reduction in the DMN, accompanied by isolated regional NVC increases in DAN and SVAN, could reflect the emergence of (defective) compensatory processes in T2DM patients in response to altered neurovascular conditions. Overall, this pattern is reminiscent of neural abnormalities previously observed in Alzheimer’s disease, suggesting that similar neurobiological mechanisms, secondary to insulin resistance and manifesting as NVC alterations, might be developing in T2DM pathology.

## Introduction

Type 2 diabetes mellitus (T2DM) is a serious metabolic chronic disease, and its complications constitute a major public health problem that affects almost all countries in the world, with high rates of related morbidity and mortality (Wu et al., 2014). Particularly, it has been observed that T2DM may accelerate the effects of aging on some brain structures, as a form of neurodegeneration, and may therefore contribute to the higher prevalence of cognitive impairment observed in T2DM patients (Abbatecola et al., 2006; Rizzo et al., 2010; Riederer et al., 2017). Emerging evidence indicates that insulin resistance and hyperinsulinemia are shared features between T2DM and Alzheimer’s disease (AD), suggesting a potential counteracting role of some antidiabetic drugs (Rizzo et al., 2014, 2022), such as also metformin, on several neurodegenerative mechanisms (Sanati et al., 2022).

Magnetic resonance imaging (MRI) has been previously used to detect structural and functional abnormalities in the T2DM brain, addressing the possible links between these alterations and some typical pathological manifestations of the disease. For example, neuropathologic studies have linked T2DM to an increased incidence of cerebral infarcts (Peila et al., 2002; Arvanitakis et al., 2006; Sarwar et al., 2010) that are usually detectable *in vivo* with MRI as white matter T1-weighted image hyperintensities (Wang et al., 2020). Moreover, other pathological states of metabolic origin occurring in T2DM (e.g., insulin resistance, hyperglycemia, and inflammation) have been associated to cerebrovascular dysfunctions (Brownlee, 2005; Zhou et al., 2014; Chung et al., 2015). However, most of the above-mentioned structural and metabolic changes have been also associated with poor cognitive performances in several functional domains, including memory, attention, and executive functions (Bangen et al., 2018; Wang et al., 2020). Indeed, the correct brain functions could be irreversibly compromised by either vascular deterioration of white matter integrity (Wang et al., 2020) or by reduced cerebral blood flow (CBF) in gray matter regions involved directly or indirectly in the cognitive functions (Bangen et al., 2018). In addition, according to epidemiological studies, T2DM and all other known risk factors for AD, are associated with a vascular component (de la Torre, 2002).

Importantly, patients with T2DM and cognitive impairments may also show decreased spontaneous brain activity on resting-state functional MRI (RS-fMRI), as indexed by amplitude of low frequency fluctuations (ALFF), regional homogeneity (ReHo) and degree centrality (DC) measures (Cui et al., 2014; Wang et al., 2014; Wang et al., 2017). These metrics have been previously used to depict different characteristics of the RS-fMRI signals regardless of the specific network substrate of the underlying functional connectivity (Zang et al., 2004; Zang et al., 2007; Zuo et al., 2012; Li et al., 2021).

However, in the last few years, the hypothesis that T2DM pathology could also provoke alterations in terms of brain neurovascular coupling (NVC), has been also formulated albeit, to the best of our knowledge, only three studies (Hu et al., 2019; Yu et al., 2019; Zhang et al., 2021) have specifically explored this phenomenon, without providing consistent or conclusive answers to the questions of (i) which specific brain regions or networks exhibit most NVC alterations in T2DM patients and (ii) whether these are related to a cognitive impairment.

In general, an NVC measure should quantify the (graded) amount of interplay existing between local cerebral perfusion and neural activity within a given region or network (Phillips et al., 2016). In practice, even if local CBF varies in space as a function of the mean local energy consumption (local metabolic demand), only part of the local neural activity covaries with it. Thus, the spatial correlation between CBF and a measure of local neural activity has been proposed as a surrogate index for NVC. Since T2DM can be associated with both CBF and fMRI signal changes during resting state conditions (RS-fMRI), it is reasonable to examine such possible NVC alterations across different RS-fMRI networks (and constituent node regions) *via* the above spatial combination of individually registered CBF and ALFF maps, as could be, respectively, derived from arterial spin labeling (ASL) and blood oxygen level dependent (BOLD) RS-fMRI non-invasive measurements.

In the current study, we present an NVC analysis according to the above model, as applied to ASL and RS-fMRI data sequentially acquired in T2DM patients (with or without cognitive impairment) and healthy volunteers, in the same MRI session. More specifically, we evaluated the group effects and the relation between NVC and cognitive performances, using region- and network-based NVC estimates, as obtained after standard preprocessing (including ALFF, ReHo and DC calculations) of BOLD RS-fMRI time-series and standard calibration and co-registration of ASL images. Because we used no *a priori* knowledge about the regions and networks potentially implicated in T2DM, we performed this analysis considering an entire standard brain parcelation as provided by the normative atlas of Schaefer et al. (2018) and all 7 RS-fMRI networks as defined for this parcelation according to the normative study by Yeo et al. (2011).

## Materials and methods

### Subjects

Eighty subjects were screened for glucose metabolism and T2DM in accordance with the diagnostic and classification criteria published by the (American Diabetes Association, 2020). For possible inclusion in the MRI study, all subjects were required to meet the following criteria: (1) being in the 55–80 age range; (2) having a minimum of 6 years of education and being able to complete the neuropsychological tests; and (3) Mini-Mental State Examination (MMSE) score >24, Clinical Dementia Rating (CDR) ≥0,5. Exclusion criteria were: (1) history of psychiatric diseases, stroke, epilepsy, brain trauma and surgery, cerebrovascular accidents, or severe liver, kidney, or heart disease, or severe hyperglycemia coma and hypoglycemia; (2) abnormal results of thyroid hormones, vitamin B12, and folate; (3) history of cardiovascular or cerebrovascular disease (Hachinski Ischemic Score ≤4), including hemorrhage, subcortical ischemic vascular disease (Liu et al., 2021); and (4) metal implants.

At the time of the MRI examination, forty-two subjects had received a diagnosis of T2DM from maximum 6 months and were either drug-naïve or taking metformin since less than 6 months. Twenty subjects, who were also screened for glucose metabolism and did not meet the criteria for T2DM proposed by American Diabetes Association (2020), were considered eligible for the study as healthy controls (HC).

Ultimately, 33 T2DM patients (age 70 ± 6 years, 15 males) and 18 HC (age 65 ± 9 years, 9 males), meeting the inclusion criteria without showing exclusions features, were enrolled. T2DM patients were either drug-naive (*n* = 19) or under treatment with metformin (*n* = 14) since less than 6 months.

This study was conducted according to the principles expressed in the Declaration of Helsinki. Ethics approval was obtained from the Ethics Committee of the University of Campania “Luigi Vanvitelli” (Prot. nr. 248/2020). Written informed consent was obtained from each participant.

### Neuropsychological assessment

All patients underwent a multi-domain, neuropsychological assessment, including mini mental state examination (MMSE), to test global cognitive performances; mental deterioration battery (MDB) (Carlesimo et al., 1996), providing eight test scores (i.e., four are expressions of the processing of verbal material and four derive from the processing of visuo-spatial material). Within MDB, the verbal tests are: Immediate and delayed recall of Rey’s 15 words (IR and DR), Word fluency (WF), Phrase construction. The visuo-spatial tests are: Raven’s 47 progressive colored matrices (PM), Immediate visual memory, Freehand copy of drawings (CD), Coping drawings with land-marks (CDL). Correction for age and education was applied to all scores. Raw scores on all neuropsychological tests were converted to age- and education-adjusted standard scores using published norms developed for the Italian population, thereby possible subgroups of T2DM patients, with (CI +) or without (CI-) cognitive impairment, were considered according to psychometric data (Carlesimo et al., 1996) and anamnestic and clinical information. The severity of CI was measured with the global clinical dementia rating (CDR), whereby CDR 0 is cognitively unimpaired, 0.5 is very mild dementia, 1 is mild dementia, 2 is moderate dementia, and 3 is severe dementia (Morris, 1993). Hamilton depression and anxiety rating scale (HDARS) scores were assessed to explore mood abnormalities and activity daily living (ADL) and instrumental activity daily living (IADL) scores were evaluated to assess functional daily performances.

### Magnetic resonance imaging data acquisition

Magnetic resonance imaging images were acquired on a 3 Tesla scanner equipped with a 32−channel parallel head coil (General Electric Healthcare, Milwaukee, WI, United States). The imaging protocol included:

(1) Three−dimensional T1−weighted sequence (gradient−echo sequence Inversion Recovery prepared Fast Spoiled Gradient Recalled−echo): repetition time (TR) = 6900 ms, echo time (TE) = 3.0 ms, voxel size 1 mm × 1 mm × 1 mm, matrix 256 × 256, inversion time (TI) = 650 ms. Duration: 6.49 min.

(2) Three-dimensional pseudo-continuous ASL (3D-PCASL) sequence: fast spin-echo with background suppression, field of view (FOV) 240 mm, slice thickness 4 mm, TR = 5306 m sec, TE = 10.5 ms, spiral read-out with 10 arms (each with 512 samples), post-labeling delay (PLD) 2525 ms (8 subjects were acquired with PLD of 2,025 ms). Duration: 5.09 min. Note: From the raw data (acquired with spiral read-out), 34 axial slices were reconstructed in-line with an effective voxel size of 3.22 mm × 3.22 mm × 4 mm. The in-line reconstruction yielded both perfusions weighted (PW) images (from the direct subtraction of label and control volumes) and proton density (PD) weighted images (with identical saturation recovery of 2,000 ms and geometry, to be used as reference for calibration).

(3) Echo-planar imaging (EPI) sequence for resting state fMRI: FOV 288 mm, matrix 96 × 96, Flip Angle (FA) 90°, TR = 1500 ms, TE = 19 ms, 320 volumes, 44 axial slices, voxel size 3 mm × 3 mm × 3 mm, slice order descending interleaved (in 5 subjects it was ascending interleaved). Duration: 8 min.

(4) Fluid−Attenuated Inversion Recovery (FLAIR) sequence: TR = 11000 ms, TE 122.7 ms, Echo train length = 18, voxel size 0.5 mm × 0.5 mm × 3 mm, anterior–posterior phase−encoding direction, number of slices 44. Fat Saturation enabled. Duration: 3.30 min.

### Magnetic resonance imaging image data analysis

We applied standard methods for the preprocessing and analysis of resting-state functional data as implemented in the Data Processing Assistant for Resting-State fMRI (DPARSF 6.0; and SPM12^1^ running on MATLAB R2015a (The Math- Works Inc., Natick, MA, United States).

Echo-planar imaging volumes were corrected for slice timing differences and head motion, band-pass filtered (0.01–0.1 Hz) and, after alignment to the anatomical T1 volumes, spatially normalized to the Montreal Neurological Institute (MNI). Last, a smoothing with a Gaussian kernel of 6 mm full width at half maximum (FWHM) was applied to the normalized functional data. To reduce the residual effects of head motion (micromotion), as well as the effects of respiratory and cardiac signals, second-order motion and physiological nuisance correction was performed using a linear regression approach. The regression model included 24 motion-related predictors (6 head motion parameter time-series, their first-order derivatives, and the 12 corresponding squared parameter time-series) and the mean time-courses from a white matter mask and a cerebrospinal fluid mask (as obtained from 3D-T1w spatial segmentation) as 2 additional predictors.

As a result, ALFF, ReHo, and DC maps were obtained in the MNI space and, then, a z-transformation was applied to all voxels belonging to a common brain mask in the same standard space and voxel resolution.

Single−subject whole−brain CBF maps were calculated from the PW and PD volumes reconstructed from the 3D-PCASL acquisition based on the calibration formula reported in Alsop et al. (2015). The resulting CBF maps were then aligned to the anatomical T1w images by applying the transformation matrix obtained from the alignment of 3D-PCASL reference volumes to the corresponding 3D-T1w images. The aligned CBF maps were then normalized to MNI space using the DARTEL procedure and the internal template derived from the DPARSF pipeline already applied to 3D-T1w and RS-fMRI data and resampled to a voxel size of 3 mm × 3 mm × 3 mm. Last, the normalized CBF maps were smoothed with a Gaussian kernel of 6 mm FWHM.

To obtain regional NVC estimates, the standard 100-region/7 networks parcelation from the Schaefer atlas (Schaefer et al., 2018) was first resampled to the 3 mm × 3 mm × 3 mm MNI space using nearest neighbor interpolation. The networks considered in this parcelation were the Visual, Somato-Motor, Dorsal Attention, Salience Ventral Attention, Limbic, Control and Default Mode Networks.

Then, for each subject, multiple NVC estimates were computed region-wise from the Pearson correlation between CBF and ALFF (NVC_ALFF–CBF_), ReHo (NVC_REHO–CBF_) and DC (NVC_DC–CBF_). The correlation *R*-values were computed across all voxels within a region of interest (ROI) or network. For statistical analysis, the *R*-values were converted to *z*-values *via* Fischer z transformation. All *z*-values were finally organized in two matrices, a 51 (subjects) × 7 (networks) matrix and a 51 (subjects) × 100 (ROIs) matrix.

A linear mixed model was applied to all matrix columns using NVC as dependent variable, group membership as independent variable of interest and age and gender as independent confounding variables (*NVC* ∼ *group* + *age* + *gender*). False discovery rate (FDR) correction was applied to the series of *p*-values expressing the statistical significance of the group membership. The conventional threshold of FDR = 5% was applied each matrix to detect ROIs or networks exhibiting statistically significant group effects.

*Post-hoc* Student’s *t*-tests were also computed in order to compare patients with and without cognitive impairment (CI + and CI- respectively) and to compare patients with metformin treatment and drug-naïve patients.

The correlation analysis between NVC and all clinical cognitive scores was performed using Spearman correlation.

## Results

Magnetic resonance imaging data from the entire sample of 33 T2DM patients and 18 HC were considered for the statistical comparisons as, for all RS-fMRI scans, the estimated motion parameters were below the thresholds used for quality assessment. The demographic and neuropsychological characterization is reported in Table 1.

**TABLE 1:** Demographic and neuropsychological characterization.

	*N*	Age	Sex F/M	Edu	MMSE	IRRW	DRRW	WF	PCM 47	CD	CDL	HADS	ADL	IADL
HC	18	65 ± 9	9/9	11 ± 3	29 ± 1	n.a.	n.a.	n.a.	n.a.	n.a.	n.a.	n.a.	n.a.	n.a.
T2 DM	33	70 ± 6	18/15	10 ± 5	27 ± 3	37 ± 8	9 ± 2	32 ± 10	24 ± 6	9 ± 2	65 ± 13	9 ± 10	6 ± 0	8 ± 1

Edu, education; MMSE, mini-mental state examination; IRRW, immediate recall of Rey’s word; DRRW, delayed recall of Rey’s word; WF, word fluency; PCM47, Raven’s 47 progressive colored matrices; CD, freehand copy of drawings; CDL, copy of drawings with landmarks; HDAS, Hamilton depression and anxiety scales; ADL, activity daily living; IADL, instrumental activity daily living.

For the NVC analysis, all three matrices were analyzed: NVC_ALFF–CBF_, NVC_REHO–CBF_, NVC_DC–CBF_ according to the RS-fMRI metric that has been used for the NVC computation. Furthermore, based on psychometric data, 7 T2DM patients were found to be cognitively impaired (CI +) whereas the remaining 26 T2DM patients were cognitively unimpaired (CI-).

**FIGURE 1:**
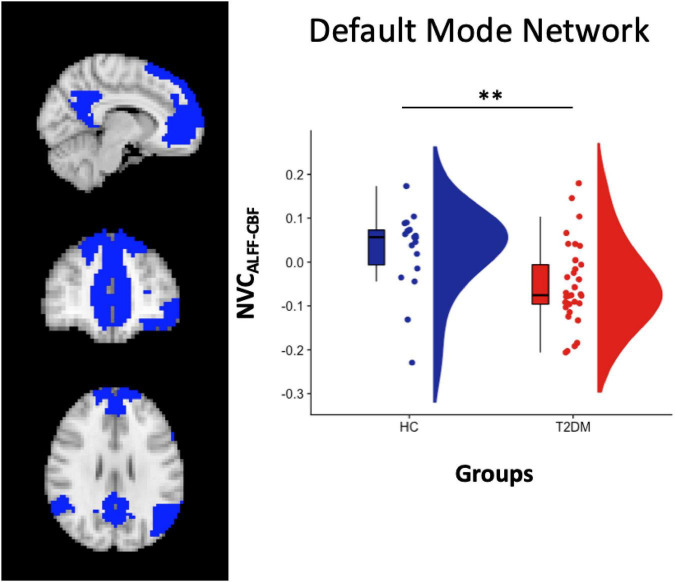
Left panel: DMN atlas representation. Right Panel: box and cloud plots for the NVC distribution in the DMN. **p < 0.01 (*p* = 0.006).

From the statistical analysis performed across the 7 resting-state networks, reduced NVC_ALFF–CBF_ was observed in T2DM patients compared to HC subjects in the DMN (Figure 1). No significant differences were found for NVC_REHO–CBF_ and NVC_DC–CBF_.

When separating patients in CI + and CI- subgroups, significant differences were found when comparing NVC_ALFF–CBF_ in both HC vs. CI- (*p* = 0.02, two-sample *t*-test) and HC vs. CI + (*p* = 0.03, two-sample *t*-test), but not in CI + vs. CI- (*p* = 0.41, two-sample *t*-test).

**FIGURE 2:**
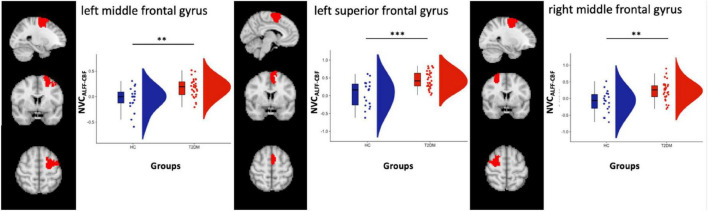
Box and cloud plots for the NVC distribution in three regions: left middle frontal gyrus, left superior frontal gyrus and right middle frontal gyrus. **p < 0.01 and ***p < 0.001 (the precise *p*-values are, respectively, *p* = 0.0014, *p* = 0.0003, and *p* = 0.0015).

From the analysis performed on the 100 ROIs, increased values of NVC_ALFF–CBF_ were found in three regions located, respectively, in the left dorsal attention network (DAN) [label 23, LH Dorsal Attention Network Frontal Eyes Field 1 in the atlas, left middle frontal gyrus, (x y z) = (−26 −3 59)], left salience ventral attention network (SVAN) [label 30, LH Salience Ventral Attention Medial 3 in the atlas, left superior frontal gyrus, (x y z) = (−6 4 62)] and in the right DAN [label 73, RH Dorsal Attention Frontal Eyes Field 1 in the atlas, right middle frontal gyrus, (x y z) = (28 −3 59)], Figure 2.

Significant differences were also found when comparing NVC_ALFF–CBF_ in HC vs. CI- (*n* = 26) in all three regions (*p* = 0.0008, *p* = 0.0002 and *p* = 0.0007, respectively, two-sample *t*-test) and HC vs. CI + (*n* = 7) in one region within the left SVAN (*p* = 0.01, two-sample *t*-test). No differences were found significant when comparing NVC_ALFF–CBF_ in CI + vs. CI- (*p* = 0.052, *p* = 0.11, and *p* = 0.27, respectively, two-sample *t*-test).

**FIGURE 3:**
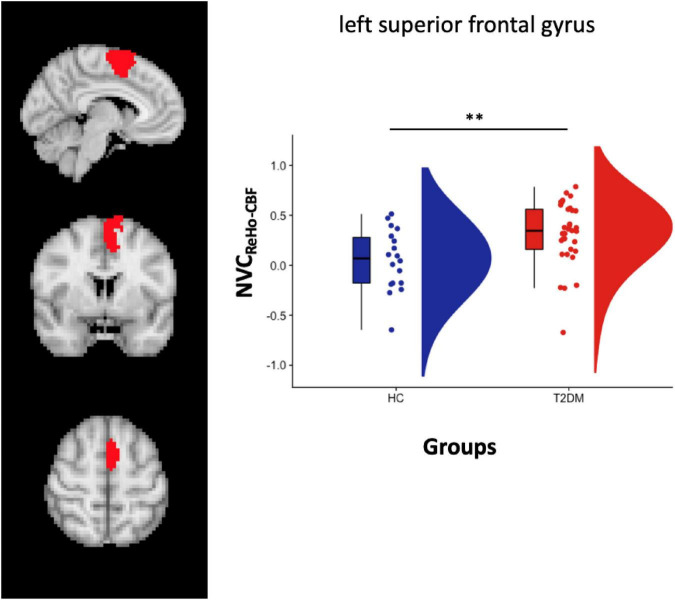
Left panel: Salience VAN atlas representation. Right Panel: box plot with points location and distribution in the Salience VAN. **p < 0.01 (*p* = 0.006).

Increased NVC_REHO–CBF_ was found in the same region located in the left SVAN [label 30, LH Salience Ventral Attention Medial 3, left superior frontal gyrus, (x y z) = (−6 4 62)] (label 30) (Figure 3). After splitting the group of patients in CI + (*n* = 7) and CI- (*n* = 26) subgroups, a significant difference was only found when comparing NVC_REHO–CBF_ in HC vs. CI- (*p* = 0.0009).

No significant differences were found across all ROIs for NVC_DC–CBF_.

When we compared the NVC measures comparing drug-naïve and metformin treated patients, no significant differences were found in any of the considered ROIs (NVC_ALFF–CBF_ in the DMN *p* = 0.26, NVC_ALFF–CBF_ in the left middle frontal gyrus *p* = 0.75, NVC_ALFF–CBF_ in the left superior frontal gyrus *p* = 0.56, NVC_ALFF–CBF_ in the right middle frontal gyrus *p* = 0.17, NVC_REHO–CBF_ in the left superior frontal gyrus *p* = 0.06).

When correlating altered NVC estimates (either NVC_REHO–CBF_ or NVC_REHO–CBF_) in T2DM patients to all collected neuropsychological and functional scores (Table 1), no significant associations emerged after Bonferroni correction for the multiple statistical comparisons. More descriptively, at an uncorrected level (*p* < 0.05), NVC_*REHO*–*CBF*_ values for left SVAN were positively correlated to age- and education-corrected scores on the cognitive task “copy of drawings,” both freehand (CD*: rho = 0.42, *p* = 0.02) and with landmarks (CDL*: rho = 0.39, *p* = 0.02), whereas NVC_*ALFF*–*CBF*_ values for the DMN were positively correlated to ADL scores (rho = 0.42, *p* = 0.02). For these scores (CD*, CDL*, ASL), the effect size (Spearmann’s rho) and the (uncorrected) *p*-value are shown in Table 2 for the NVC estimates that were found altered in the large-scale networks of T2DM patients.

**TABLE 2:** Correlation values (rho, p) of NVC estimates (altered in T2DM patients) with CD (freehand copy of drawings), CDL (copy of drawings with landmarks) and ADL (Activity Daily Living).

Regions	CD*		CDL*		ADL	
			
	rho	p	rho	p	rho	*p*
L-DAN (ALFF-CBF)	0.07	0.69	0.04	0.83	−0.11	0.54
L-SVAN (ALFF-CBF)	0.28	0.12	0.20	0.26	−0.31	0.09
R-DAN (ALFF-CBF)	−0.07	0.68	0.25	0.17	−0.34	0.06
L-SVAN (REHO-CBF)	0.42	0.02	0.39	0.02	−0.03	0.88
DMN (ALFF-CBF)	−0.03	0.88	−0.15	0.41	0.42	0.02

*Age- and education-corrected scores.

## Discussion

In this work, we performed a network- and region-based NVC analysis in a group of T2DM patients, with or without clinically relevant cognitive impairment. Starting from multiple NVC estimates, as derived from the spatial combination of CBF and RS-fMRI brain maps, we were able to report a specific pattern of NVC alterations in TD2M patients, as resulting from the comparisons of NVC measures between T2DM patients and HC groups. In addition, we further explored, both the potential role of the current neurological state in explaining such differences and the association between altered NVC and cognitive performances in T2DM patients. Here we reported a series of NVC findings, which, albeit only preliminary, may help gathering new hypotheses on the potential neurological impact of T2DM pathology.

Among positive findings, from the network-based analysis, we found that NVC_*ALFF_CBF*_ measure for the DMN was significantly reduced in T2DM patients, both as a whole group and when differentiated in those with and without cognitive impairment, compared to HC subjects.

While in HC the mean NVC is positive, in T2DM patients the mean NVC resulted negative, and the change of sign is a key point of the result. Indeed, while generally densely connected areas, such as DMN areas, exhibited high CBF, indicating tight coupling between perfusion and brain functional connectivity at rest and suggesting that highly connected functional hubs are more metabolically active and require higher levels of energy consumption (Galiano et al., 2020), this behavior is altered in people with T2DM diabetes, where a balance between the local connectivity and the obtained metabolic supply is probably disrupted.

The vulnerability of the DMN is a well-established finding in AD, corroborated several previous studies reporting evidence of selective and progressive functional connectivity disruption within this network in AD patients, starting in early stages of mild cognitive impairment and preclinical stages (Greicius et al., 2004; Sorg et al., 2007; Lim et al., 2014; Zhou et al., 2014; Nuttall et al., 2016; Pasquini et al., 2017). Thereby, the NVC reduction observed in the DMN of T2DM patients would both explain the similarity with AD observed in the neurological manifestations and suggest that this pathological condition might be facilitating the progression of neurodegeneration by affecting the brain at the level of neurovascular units, i.e., where neuronal, glial and vascular cells, among other functions, couple together to regulate the microvascular blood flow in the brain (Lo and Rosenberg, 2009; Quelhas et al., 2019), to preserve the homeostasis of the central nervous.

However, the ROI-based analysis also revealed increased NVC_*ALFF_CBF*_ in T2DM patients, compared to HC subjects, in two regions within the left and right DAN, and in one region within the left SVAN. In these regions, albeit no significant differences emerged for NVC_*ALFF_CBF*_ from the comparison between cognitively impaired and cognitively unimpaired patients, two different NVC measures, i.e., NVC_*ALFF_CBF*_ and NVC_*REHO_CBF*_, were found increased in T2DM patients, compared to HC subjects. In addition, NVC_*REHO_CBF*_ in SVAN regions was positively correlated to the cognitive performances of T2DM patients in some visuo-spatial tasks (i.e., freehand copy of drawings and coping drawings with landmarks) and NVC_*ALFF_CBF*_ in DMN regions were found positively correlated to ADL scores.

Altogether, these findings underline opposite patterns of NVC alterations in DMN vs. (bilateral) DAN and (left) SVAN in patients with early diagnosis of T2DM, independently of cognitive impairment. More specifically, despite a widespread (AD-like) NVC impairment of the DMN, NVC is also increased in other cognitive networks, such as DAN and SVAN (i.e., anterior insular, and inferior parietal cortices), well known to be, respectively, involved in executive and emotional functions and in the processing of visual stimuli. This latter involvement is also characterized by significant correlations between NVC_*ALFF_CBF*_ measures in (regions) of DAN and left SVAN and performances at copy of drawing, suggesting that a peculiar form of brain functional plasticity might be occurring, triggered by the homeostatic response of the central nervous system.

Undoubtedly, a peculiar and interesting aspect of our results is the opposite trend in the NVC alterations emerging from the group comparisons across different cognitive networks: in fact, in the DMN, we observed decreased NVC_ALFF–CBF_ in patients compared to HCs, whereas, in the DAN and in the SVAN, T2DM patients showed increased NVC_ALFF–CBF_ and NVC_REHO–CBF_ compared to HC.

In general, following the three-domain model of Menon (2011) to explain large-scale network pathology, we would be prone to believe that opposite trends in the network integrity would be required to T2DM patients for the maintenance of the intrinsic balance between functionally connected regions. Within this general framework, our findings of co-occurring increased and decreased NVC effects in T2DM would add evidence to the facts that (i) NVC plays a critical role in balancing the network (spontaneous) fluctuations between the two cognitive modes in T2DM and (ii) specific node regions in the attention domain are likely consuming more energy in T2DM patients to sustain the balance of these fluctuations. Thus, as far as we can interpret the opposing neural changes occurring at the larger scale of multiple networks as a distributed compensatory response of the brain to defective NVC, the local increase in NVC_ALFF–CBF_ in areas of one specific network could represent a sign that the energy consumption is (pathologically) unbalanced across domains. Particularly, the increased NVC in the frontal regions (of both the DAN and SVAN networks) observed in T2DM patients, could reflect the emergence of compensative circuits in the brain of T2DM patients, to specifically counteract the AD-like neurodegeneration known to affect the posterior part of the DMN (Zhou et al., 2010). In this regard, our findings may recall the recent theory of a neurobiological link between AD and insulin resistance, showing overlapping brain abnormalities between the 2 conditions (Abbatecola et al., 2006; Rizzo et al., 2010; Lynn et al., 2022). However, in our sample of T2DM patients, the potential effect of metformin, anti-diabetic drug counteracting insulin resistance, hypothesized to limit neurodegenerative changes of the brain (Sanati et al., 2022) was not confirmed, considering that no significant differences in NVC abnormalities were found by comparing drug-naïve patients to those taking metformin, most likely due to the fact that the latter subgroup included both a small patients number and patients who had started the pharmacological therapy since less than 6 months.

As the observed alteration of neuroimaging signals may be due to the primary effects of diabetes on CBF (independent of neural functions), it is worth mentioning that the NVC can be thought of as a mechanism for modulating perfusion according to the neuro-metabolic demands at the cellular level. At this level, the so-called neurovascular unit, which consists of a set of interacting neurons (pyramidal cells and interneurons), astrocytes, smooth muscle cells, endothelial cells, and pericytes, ultimately restores the adequate blood supply for the regional neural activity and metabolic demand under normal conditions (Iadecola, 2017). However, generally, one of the adverse effects of NVC is hyperglycemia, i.e., the increase of glucose concentrations that determines oxidative stress contributing to endothelial and microvascular dysfunction ultimately causing neurovascular uncoupling (Park et al., 2007). In fact, for example, it has been reported that endothelial cells acquire a senescent phenotype upon exposure to high glucose concentration and to other conditions that manifest in presence of metabolic disturbances (e.g., inflammation), and, consequently, this aspect also contributes to developing NVC alterations (Phoenix et al., 2022). These conditions are extremely common in T2DM and may partly explain the NVC abnormalities of NVC observed in our population (Barloese et al., 2022).

To the best of our knowledge, only three studies, so far, have investigated the potential impairment of NVC in T2DM patients (Hu et al., 2019; Yu et al., 2019; Zhang et al., 2021), but, because several non-overlapping regions were reported as altered across these studies, even merging our results with previous evidence would allow no definite statement about where to specifically expect the most pronounced NVC alterations in the brain of T2DM patients.

In the most recent study (Zhang et al., 2021), decreased NVC was reported in the left insula, left post-central gyrus, in the right rolandic operculum and in the right precentral gyrus in T2DM patients, whereas, in the earlier study by Yu et al. (2019), T2DM patients without cognitive impairment displayed significant NVC decrease in several regions belonging to the DMN. Finally, in the study by Hu et al. (2019), alterations in NVC were also reported in several regions, including left hippocampus, fusiform gyrus, right putamen, right middle frontal gyrus, middle frontal gyrus (orbital part), left amygdala, left superior temporal and right middle temporal gyrus and in nucleus pallidum, and in the median cingulate and paracingulate gyri.

Here, for the first time, we report NVC alterations in T2DM patients across large-scale cognitive networks using a specific brain functional parcelation, such as the one provided by Schaefer et al. (2018), where both individual regions and networks had been previously defined (and linked to networks) according to a rigorous criterion of spatial homogeneity and temporal similarity of RS-fMRI signals using data from a large sample of healthy subjects. Overall, our results seem to capture NVC alterations in brain areas that were reported in previous studies, e.g., in the DMN, as in Yu et al. (2019) or in frontal regions like Hu et al. (2019).

In the light of the hypothesized overlapping pathogenetic mechanisms existing between AD and T2DM, we firstly considered a simple splitting of the T2DM group into CI + and CI- subgroups. However, none of the above discussed observed alterations could be explained by the sole presence (or absence) of cognitive impairment, since no significant differences between CI + and CI- groups were found across all networks and regions. On the other hand, we acknowledge that this might be also due to the extremely limited sample size of the CI + subgroup, that might call for future studies over much larger samples of T2DM patients.

Nonetheless, considering that T2DM has been previously associated with reduced performances across multiple cognitive domains (Zilliox et al., 2016), we secondly proceeded with correlating each of the (altered) NVC estimates to several clinical scores across all individual T2DM patients, regardless of the general CI condition. While we found a significant positive correlation between visuo-spatial abilities (ADL scores) and NVC measures in (regions of) the DAN and the left SVAN, as discussed previously, even in this case, the statistical power has been certainly affected by the relatively small sample size of the patients studied.

Aside from NVC, alterations in the DMN of T2DM patients have been previously observed in separate RS-fMRI and CBF studies, reporting either reduced functional connectivity [see, e.g., (Marder et al., 2014; Chen et al., 2016)] or reduced regional CBF (Wang et al., 2021). On the other hand, altered functional connectivity in T2DM has been also reported within the attentional networks [see, e.g., (Xia et al., 2015)], albeit the proper framing of these neural effects within a more large-scale dysfunctional interaction between the DAN and DMN in T2DM patients has been only recently hypothesized by Lei et al. (2021) based on the observed differential pattern of inter-network functional connectivity existing between T2DM and HC groups.

Overall, these results appear complementary with the NVC results highlighted here. In fact, while Lei et al. (2021) were able to relate the DAN-DMN anti-correlation to the poor modulation of attentional processes in response to shifting cognitive demands, here we added the notion that such an unbalance in the neural resource management could be linked to defective NVC within and across the two networks, as discussed above. Thus, as far as future studies will confirm the neurovascular origin of this unbalance, it would be more than likely that these neural effects can occur in the earliest stages of cognitive decline, i.e., before the cognitive impairment becomes manifest at the clinical evaluation (Gorelick et al., 2016; Cheng et al., 2021; Huang et al., 2021; Liu et al., 2021).

One last observation concerns the different metrics used for NVC estimates. As all metrics are surrogated from the same RS-fMRI data, like most previous NVC studies, here we included results from all three NVC metrics, albeit most of the positive findings reported here (and previously) were related to the coupling of ALFF and CBF estimates (NVC_ALFF–CBF_). This was not surprising considering that ALFF is the most local measure of neural activity that can be obtained from the RS-fMRI signal at the level of single voxels (Zang et al., 2007), thereby, it is expectedly the most sensitive measure to the microvascular defects that eventually developed within the neural tissue.

Nonetheless, only the NVC estimates from the ReHo metric, which is an index of neural synchronization between a given voxel and its closest neighboring voxels (Zang et al., 2004), was able to capture highly significant NVC increases within the left SVAN, whereas the DC metric revealed no significant NVC alterations in T2DM patients. This is again not surprising since DC reflects the amount of synchronization between a given voxel and all voxels throughout the brain (Zuo et al., 2012; Li et al., 2021), discounting the fact that there might exist very specific regions (near or far from the same given voxel) highlighting a defective NVC.

This work has a limitation regarding the sequence parameters of flip angle (FA) and TE. Indeed, the first was set to 90° which is likely above the Ernst angle that determines BOLD contrast to be enhanced by inflow effects. The TE was set to 19 ms, likely below the optimal value at 3T, and further enhancing the relative contribution of inflow effects (Havlicek et al., 2017).

Nevertheless, according to recent work by Yuan et al. (2021) performed to verify the effects of different echo times on local connectivity metrics, the local activity metrics displayed different TE dependency characteristics but the overall change patterns were similar. Thus, starting from this observation, we could still assume that the patterns of local connectivity obtained in our populations are overall similar to those obtained with a higher TE. Another aspect that deserves mention is that the measures of local connectivity computed in this analysis were not used directly to compare patients and controls but the regional values of these metrics were correlated to the regional values of CBF and, thus, possible effects of inflow would have increased the obtained correlations for every subject, belonging both to the healthy control group or to the diabetes group, without affecting the final group comparison. Thus, even in presence of suboptimal parameters, it is still possible to assume that the group differences are still related to NVC alterations, e.g., different patterns of correlations between resting-state metrics and perfusion, rather than to the increased inflow effects.

In conclusion, this study reveals the presence of AD-like NVC alterations in the DMN and in multiple functional regions belonging to the bilateral DAN and to the left SVAN networks in T2DM patients. These NVC alterations were variably correlated to some clinical cognitive scores, albeit not to the clinical manifestation of cognitive impairment or to the use of metformin, in the studied T2DM patients.

Albeit preliminary, the observed NVC effects might signal an early compensating response of the central nervous system to the emergence of cerebro-vascular defects within major large-scale cognitive networks, which could pose a higher risk for cognitive decline in T2DM patients and therefore deserves further investigations.

## Data availability statement

The raw data supporting the conclusions of this article will be made available by the authors, without undue reservation.

## Ethics statement

The studies involving human participants were reviewed and approved by the Ethics Committee of the University of Campania “Luigi Vanvitelli”. The patients/participants provided their written informed consent to participate in this study.

## Author contributions

AC, FE, and MC contributed to the conception and design of the study. FT organized the database. AC performed the statistical analysis and wrote the first draft of the manuscript. FE, GT, FT, and MR wrote sections of the manuscript. All authors contributed to the manuscript revision, read, and approved the submitted version.

## Footnotes

1
http://www.fil.ion.ucl.ac.uk/spm
