# Supplementary material for: Neurovascular coupling in patients with type 2 diabetes mellitus
Source: Front Aging Neurosci. 2022 Sep 1;14:976340. doi: 10.3389/fnagi.2022.976340 (PMC9476313; doi:10.3389/fnagi.2022.976340)
Supplement: Supplementary file 1 [file Table_1.docx]

|  | MMSE | | IRRW | | DRRW | | WF | | PCM47 | | HADS | | IADL | |
| --- | --- | --- | --- | --- | --- | --- | --- | --- | --- | --- | --- | --- | --- | --- |
|  | rho | p | rho | p | rho | p | rho | p | rho | p | rho | p | rho | p |
| **L-DAN**  **(ALFF-CBF)** | 0.01 | 0.94 | 0.26 | 0.14 | -0.11 | 0.54 | 0.05 | 0.79 | -0.14 | 0.44 | -0.18 | 0.32 | -0.22 | 0.22 |
| **L-SVAN (ALFF-CBF)** | -0.29 | 0.10 | -0.03 | 0.86 | -0.17 | 0.36 | 0.12 | 0.51 | -0.06 | 0.76 | 0.00 | 0.99 | 0.21 | 0.25 |
| **R-DAN**  **(ALFF-CBF)** | 0.21 | 0.24 | 0.20 | 0.28 | 0.00 | 0.99 | 0.07 | 0.72 | 0.15 | 0.40 | -0.11 | 0.54 | 0.03 | 0.89 |
| **L-SVAN (REHO-CBF)** | -0.06 | 0.76 | -0.07 | 0.70 | -0.06 | 0.75 | 0.34 | 0.05 | 0.09 | 0.63 | -0.06 | 0.72 | 0.01 | 0.98 |
| **DMN**  **(ALFF-CBF)** | 0.05 | 0.79 | -0.19 | 0.28 | -0.03 | 0.88 | 0.10 | 0.56 | 0.02 | 0.92 | -0.17 | 0.36 | -0.25 | 0.17 |

**Supplementary Table**: Correlations with the remaining clinical scores. MMSE =mini-mental state examination IRRW=Immediate Recall of Rey’s word, DRRW=Delayed Recall of Rey’s word, WF = Word Fluency, PCM47=Raven's 47 progressive colored matrices, HDAS=Hamilton depression and anxiety scales, IADL = instrumental activity daily living.
